# CUL4A overexpression enhances lung tumor growth and sensitizes lung cancer cells to Erlotinib via transcriptional regulation of EGFR

**DOI:** 10.1186/1476-4598-13-252

**Published:** 2014-11-21

**Authors:** Yunshan Wang, Pengju Zhang, Ziming Liu, Qin Wang, Mingxin Wen, Yuli Wang, Hongtu Yuan, Jian-Hua Mao, Guangwei Wei

**Affiliations:** Department of Anatomy and Key Laboratory of Experimental Teratology, Ministry of Education, Shandong University School of Medicine, 44 Wenhua Xi Road, Jinan, Shandong 250012 P.R. China; Department of International Biotechnology R&D Center, Shandong University School of Ocean, 180 Wenhua Xi Road, Weihai, Shandong 264209 P.R. China; Department of Biochemistry and Molecular Biology, Shandong University School of Medicine, 44 Wenhua Xi Road, Jinan, Shandong 250012 P.R. China; Department of Neurosurgery, The Fifth People’s Hospital, 447 Jingshen Road, Jinan, 250022 P.R. China; Department of Anesthesiology, Qilu Hospital, Shandong University, 107 Wenhua Xi Road, Jinan, 250012 P.R. China; Department of Pathology, Shandong Cancer Hospital and Institute, Jinan, 250012 P.R. China; Life Sciences Division, Lawrence Berkeley National Laboratory, Berkeley, CA 94127 USA

**Keywords:** CUL4A, Lung cancer, EGFR, Erlotinib

## Abstract

**Background:**

CUL4A has been proposed as oncogene in several types of human cancer, but its clinical significance and functional role in human non-small cell lung cancer (NSCLC) remain unclear.

**Methods:**

Expression level of CUL4A was examined by RT-PCR and Western blot. Forced expression of CUL4A was mediated by retroviruses, and CUL4A silencing by shRNAs expressing lentiviruses. Growth capacity of lung cancer cells was measured by MTT *in vitro* and tumorigenesis *in vivo*, respectively.

**Results:**

We found that CUL4A was highly expressed in human lung cancer tissues and lung cancer cell lines, and this elevated expression positively correlated with disease progression and prognosis. Overexpression of CUL4A in human lung cancer cell lines increased cell proliferation, inhibited apoptosis, and subsequently conferred resistance to chemotherapy. On other hand, silencing CUL4A expression in NSCLC cells reduced proliferation, promoted apoptosis and resulted in tumor growth inhibition in cancer xenograft model. Mechanistically, we revealed CUL4A regulated EGFR transcriptional expression and activation, and subsequently activated AKT. Targeted inhibition of EGFR activity blocked these CUL4A induced oncogenic activities.

**Conclusions:**

Our results highlight the significance of CUL4A in NSCLC and suggest that CUL4A could be a promising therapy target and a potential biomarker for prognosis and EGFR target therapy in NSCLC patients.

**Electronic supplementary material:**

The online version of this article (doi:10.1186/1476-4598-13-252) contains supplementary material, which is available to authorized users.

## Background

Lung cancer remains by far the most common cause of cancer mortality and non-small cell lung cancer (NSCLC) accounts for >80% of cases of lung cancer, which ranks among the most deadly cancers worldwide [1]. Although three therapeutic modalities (surgical resection, chemotherapy, and radiotherapy) have been established, long-term survival for lung cancer patients is still generally poor [1, 2]. Therefore, further characterization of NSCLC pathogenesis to identify useful biomarkers and explore novel therapeutic targets becomes an essential task.

Epidermal growth factor receptor (EGFR) is a transmembrane protein with intrinsic tyrosine kinase activity that regulates cell growth in response to binding of its ligands. EGFR is overexpressed or mutated in most NSCLC cases, and deregulated expression of EGFR together with ligand binding and concomitant receptor activation promotes tumor cell growth, proliferation, and survival [3, 4]. Several studies have demonstrated that EGFR overexpression correlates with reduced disease-free and overall survival [5, 6]. Therefore, many strategies including using specific tyrosine kinase inhibitors (TKI) and monoclonal antibodies to target EGFR have been developed for treatment of NSCLC [7, 8].

CUL4A, a member of the cullin family of proteins that composes the multifunctional ubiquitin ligase E3 complex, plays critical roles in DNA replication, cell cycle regulation and genomic instability [9–15]. CUL4A amplification or overexpression has been reported in some human cancers, including breast cancer, squamous cell carcinoma, adrenocortical carcinoma, childhood medulloblastoma, prostate cancer and hepatocellular carcinoma and is associated with poor prognosis in node-negative breast cancer [16–23]. Recently, it has benn shown that CUL4A is overexpressed and amplified in 64% primary malignant pleural mesothelioma, and downregulation of CUL4A with shRNA causes cell cycle arrest and growth inhibition through upregulation of p21 and p27 proteins [20]. The use of a Cul4A transgenic mouse model demonstrates the potential oncogenic role of Cul4A in lung tumor development. After 40 weeks of Cul4A overexpression, lung tumors were visible and were characterized as grade I or II adenocarcinomas [24]. Kim *et al*. reported that DLC1 was ubiquitinated and degraded by cullin 4A-RING ubiquitin ligase (CRL4A) complex interaction with DDB1 and the FBXW5 substrate receptor in NSCLC cell lines [25]. The recently report also shown that EGFR protects proliferating cell nuclear antigen from cullin 4A protein-mediated proteolysis [26]. However, the functions and mechanism of CUL4A in NSCLC development and progression remain largely unknown.

In the present work, we sought to investigate the role and mechanism of CUL4A in NSCLC. We first examined both mRNA and protein expression patterns and evaluated prognostic significance of CUL4A in NSCLC. High levels of CUL4A predicted poor prognosis in overall survivals. Moreover, ectopic expression of CUL4A promoted cell proliferation and inhibited apoptosis. Knockdown of endogenous CUL4A by shRNA significantly decreased cell proliferation and tumorigenesis. Those oncogenic functions of CUL4A are at least partially mediated by regulation of EGFR and its related pathways. Additionally, we showed that CUL4A overexpression conferred NSCLC cells resistance to chemotherapy and sensitivity to EGFR target therapy. Our findings implicate CUL4A as a promising molecular target for therapy and a prognostic marker for highly recurrent NSCLC.

## Results

### CUL4A expression is high and associated with prognosis in lung cancer

**Figure 1:**
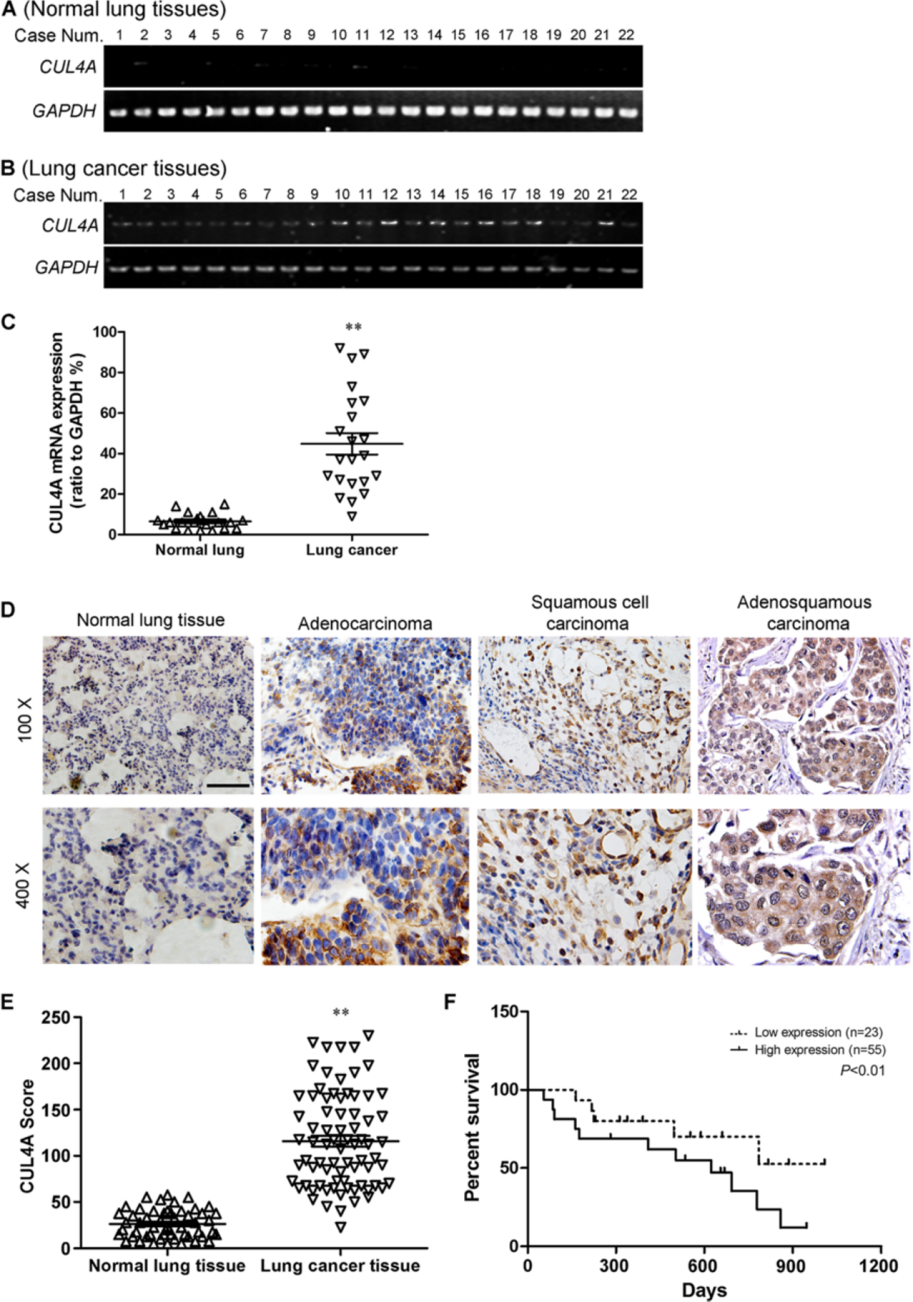
**CUL4A is overexpressed and associated with prognosis in lung cancer. (A)** RT-PCR analysis of CUL4A mRNA in normal lung tissues (n =22). **(B)** RT-PCR analysis of CUL4A mRNA in lung cancer tissues (n =22). **(C)** Relative mRNA levels of CUL4A (normalized to GAPDH) in normal lung tissues and lung cancer tissues were shown as scatter diagram. **(D)** Immunohistochemistry analysis of CUL4A protein levels in normal lung tissues and NSCLC specimens of different subtypes. **(E)** CUL4A expression scores in normal lung tissues and lung cancer tissues. **(F)** Survival curves of NSCLC patients with low versus high expression of CUL4A (n =78; *P* <0.01, log-rank test). Scale bar indicates 50 μm **(D)**. ^**^ *P* <0.001 *vs* normal lung tissues based on Student’s *t*-test. Experiments in A-B were repeated three times. Error bar indicate standard deviation.

We first examined CUL4A expression in a panel of 7 human lung cancer cell lines and 2 normal human lung epithelial cell lines. RT-PCR (Additional file 1: Figure S1A) and Western blot (Additional file 1: Figure S1B) showed high level of CUL4A in nearly all of tumor cell lines compared with normal human lung epithelial cells. We then determined CUL4A expression in clinical samples using RT-PCR. Of 22 NSCLC patients, 18 (81.8%) had higher CUL4A mRNA levels than adjacent normal lung tissues (Figure 1A and B). Overall, the average CUL4A mRNA levels in the cancer tissues were significantly higher than that in the normal lung tissues (*P* <0.001, Figure 1C). Moreover, we performed immunohistochemistry analysis in 78 NSCLC specimens and 56 normal lung tissues and found that CUL4A level was higher in 87.2% of tumor samples (68 of 78) than that in normal lung tissue. The CUL4A protein appeared to be expressed in both cytoplasmic and nuclear components of tumor cells with stronger signal observed in cytoplasm (Figure 1D). While the normal bronchial epithelia exhibited undetectable or low CUL4A staining (Figure 1E).

To evaluate the prognostic value of CUL4A expression in NSCLC, we divided the NSCLC patients into CUL4A high and low expression groups based on a cutoff score of 73. Survival analysis revealed that NSCLC patients with high CUL4A expression had poorer overall survival than those with low CUL4A expression (*P* <0.01; Figure 1F). Next, we analyzed the relationship between CUL4A expression levels and clinicopathological characteristics. CUL4A expression was not correlated with gender, age or tumor subtype (Table 1) but statistically significantly correlated with NSCLC clinical stages (Table 1). All together, we demonstrated that CUL4A is overexpressed in NSCLC and high level of CUL4A expression is a prognostic predictor of progression and poor clinical outcome in NSCLC patients.

**Table 1:** **Correlation between the clinical pathologic features and expressions of CUL4A**

Characteristics		CUL4A		***P***-value ^a^
		Low or None	High	
Gender	Male	21	29	0.732
	Female	13	15	
Age (years)		53.7 ± 11.6	62.2 ± 15.3	0.197
Pathology	Squamous cell carcinoma	14	16	0.249
	Adenocarcinoma	11	18	
	Adenosquamous carcinoma	9	10	
Clinical stage	I	12	5	<0.01^b^
	II	9	10	
	III	8	17	
	IV	5	12	

^a^ *X* ^2^ test.

^b^Comparing clinical stages I versus II-IV.

### CUL4A regulates NSCLC cell growth and tumorigenesis

**Figure 2:**
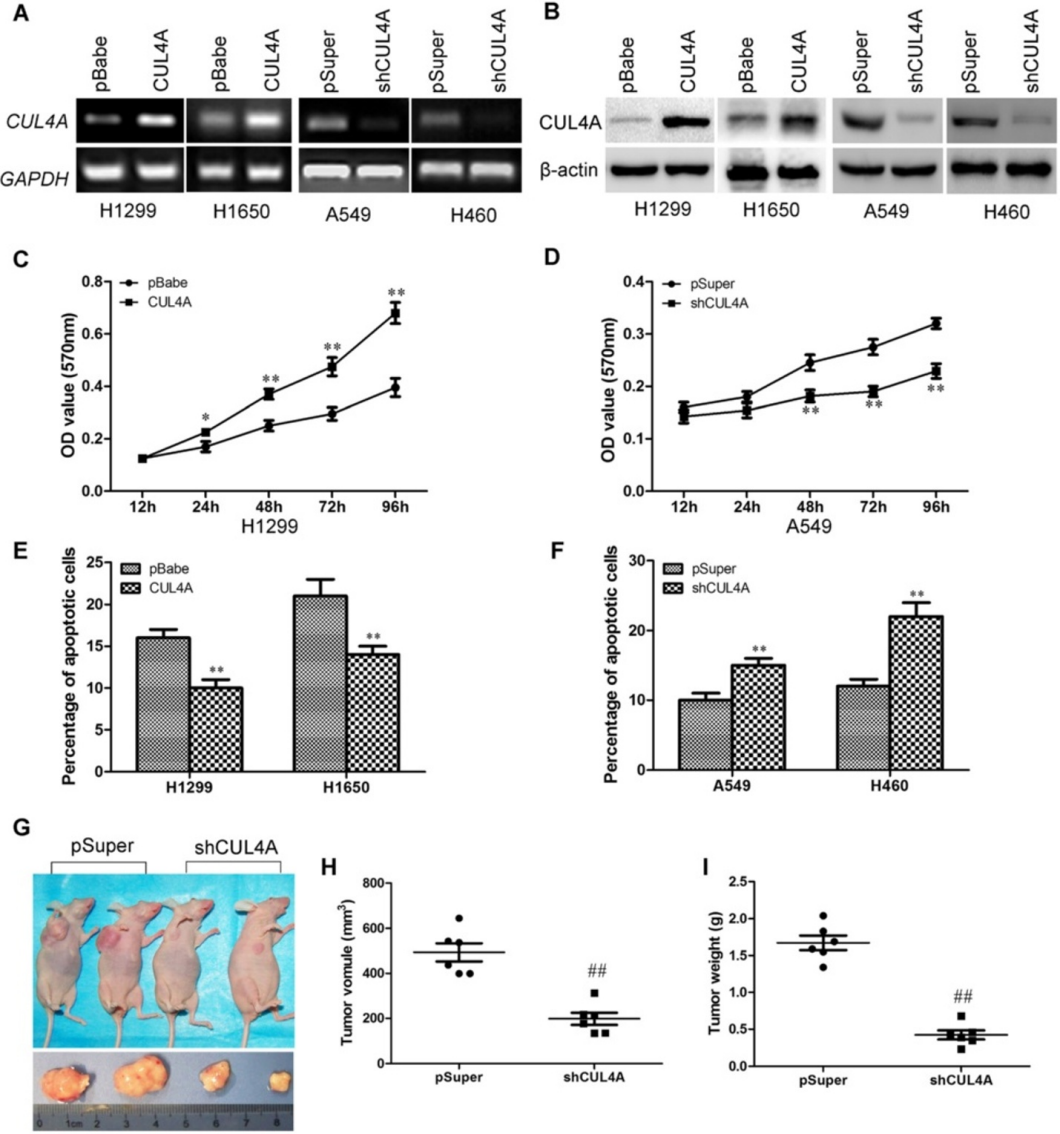
**CUL4A regulates NSCLC cell growth both** ***in vitro*** **and** ***in vivo*** **.** Ectopic and silencing CUL4A expression in H1299, H1650, A549 and H460 cells were established by viral transduction. The levels of CUL4A in these resultant cell lines were verified by RT-PCR **(A)** and Western blot **(B)**. Cell proliferation *in vitro* was examined by MTT **(C and D)**. Apoptosis was estimated using Annexin V staining as described in Methods **(E and F)**. Tumorigenic capacity of A549 and A549-shCUL4A cells was assess *in vivo* (**G**, **H**, and **I**, *n* =6). ^*^ *P* <0.05 and ^**^ *P* <0.01 *vs* pBabe cells; ^#^ *P* <0.05 and ^##^ *P* <0.01 *vs* pSuper cells. All results in A to F are from three independent experiments. Error bar indicate standard deviation.

In order to test the oncogenic activity of CUL4A in NSCLC, H1299 and H1650 cells were used to establish CUL4A overexpressing cell lines and A549 and H460 cells were used to establish CUL4A silencing cell lines by viral transduction. The levels of CUL4A in these resultant cell lines with forced CUL4A expression (designated as H1299-CUL4A and H1650-CUL4A) and silenced CUL4A expression (designated as A549-shCUL4A and H460-shCUL4A) were verified by RT-PCR (Figure 2A) and Western blot (Figure 2B).

We then used these cell lines to assess the effect of CUL4A on cell growth by MTT assay. Both H1299-CUL4A and H1650-CUL4A cell lines had a significant increase in cell proliferation compared with their respective controls, in contrast, A549-shCUL4A and H460-shCUL4A cell lines had lower rates of cell proliferation (Figure 2C and D, Additional file 2: Figure S2A and S2B). To test whether CUL4A overexpression regulates lung cancer cells transformation, we examined anchorage-independent cell growth by soft agar colony formation assay. Numbers of colonies formed by H1299-CUL4A were significantly higher than those by pBabe control cells (Additional file 3: Figure S3A), while the numbers of colonies formed by A549-shCUL4A were significantly lower than those by pSuper control cells (Additional file 3: Figure S3B).

To further understand and characterize the role of CUL4A in control of NSCLC cell growth, we analyzed the apoptotic activity of CUL4A in NSCLC cells. Annexin V binding assay showed that ectopic CUL4A expression reduced the cell proportion in apoptosis and silencing CUL4A expression drastically increased the population of apoptotic cells (Figure 2E and F).

To extend our *in vitro* observations, we investigated whether CUL4A could regulate tumorigenic capacity of NCSLC cells *in vivo*. A549-shCUL4A and its corresponding control cells were subcutaneously injected into nude mice. Tumor size was measured every other day up to 40 days. As expected, the tumors from A549-shCUL4A cells grew less rapidly at the implantation site than its control cells. After 40 days, tumors were collected and the shCUL4A tumors had a smaller size compared to the pSuper (shCUL4A tumors load to be ~40% of the size of the pSuper tumors) (Figure 2G and H). Consistent with these observations, the expression of major proliferation related protein, Ki67, was modulated upon CUL4A expression, silencing CUL4A dramatically decreased the expression levels of Ki67 (Additional file 4: Figure S4). Taking together, these results suggest that CUL4A is an important regulator of proliferation in lung cancer cells in vivo.

We then analyzed if CUL4A affect the sensitivity of NSCLC cells to chemotherapy, H1299 and H1650 cells with overexpression or A549 and H460 cells with silence of CUL4A were treated with various doses of docetaxel and doxorubicin. H1299-CUL4A and H1650-CUL4A cells displayed significantly higher survival rates than the vector control cells after treatment for 48 h, whereas the number of dead cells markedly increased when CUL4A expression was silenced by specific shRNA (Additional file 5: Figure S5A-H). These results indicate that CUL4A overexpression confers docetaxel and doxorubicin resistance in lung cancer cells.

### CUL4A regulates EGFR transcriptional expression

**Figure 3:**
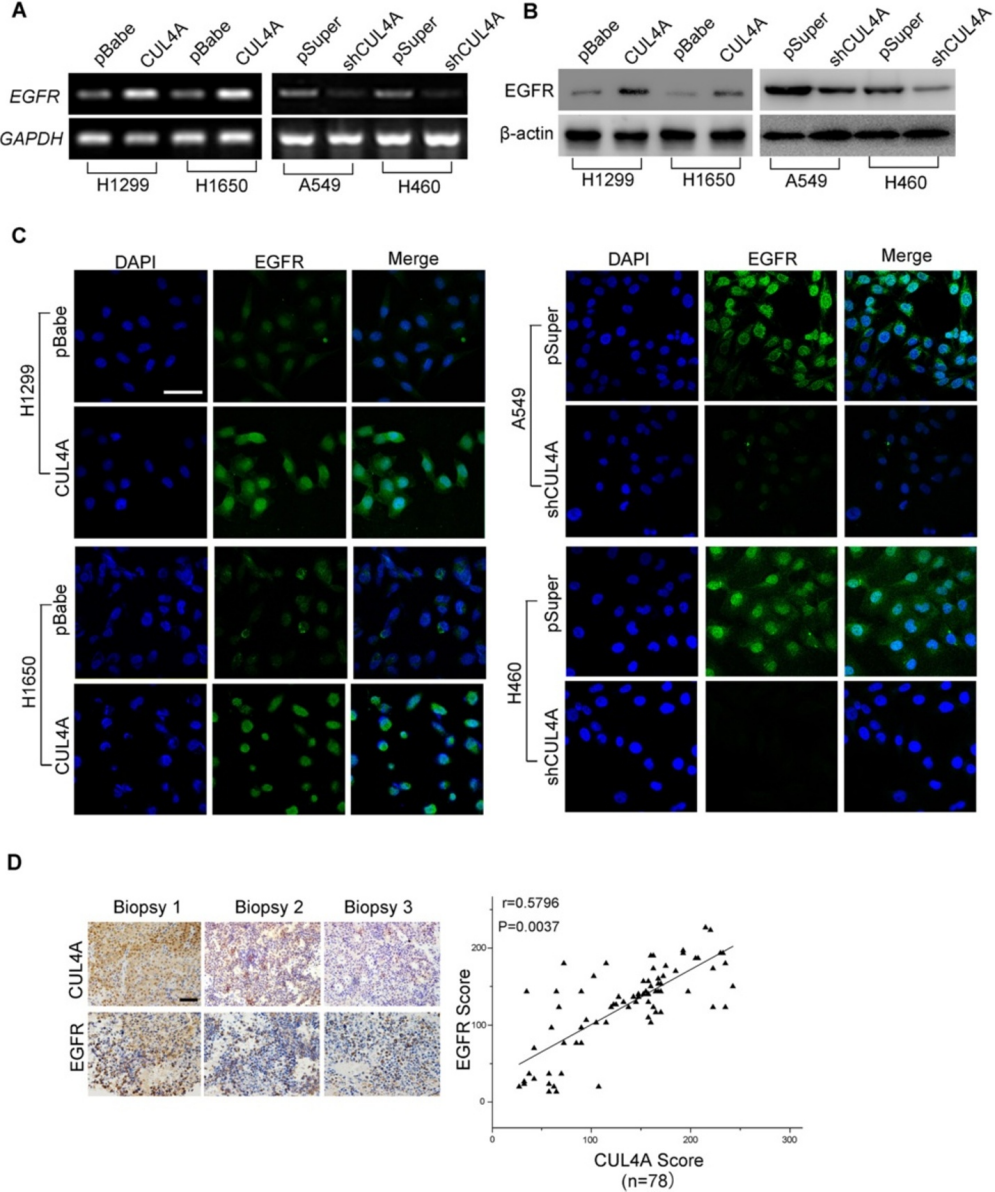
**CUL4A regulates EGFR expression. (A)** RT-PCR analysis of the expression of EGFR mRNA in H1299, H1650, A549 and H460 cells. **(B)** Western blot analysis of the expression of EGFR protein in H1299, H1650, A549 and H460 cells. **(C)** Immunofluorescence microscopy analysis of EGFR expression of in H1299, H1650, A549 and H460 cells. **(D)** The immunohistochemistry analysis of CUL4A and EGFR expression in NSCLC biopsy showed that CUL4A levels significantly correlate with EGFR levels in NSCLC tissues. All results are from three independent experiments. Scale bar indicates 20 μm **(C)**, and 50 μm **(D)**.

As EGFR is overexpressed in NSCLC cells and plays a key role in the control of cell growth [27], to elucidate the mechanism by which CUL4A regulates cell growth in NSCLC, we investigated the effect of CUL4A on EGFR expression. CUL4A overexpression significantly increased the level of EGFR transcript, while suppression of CUL4A dramatically decreased the level of EGFR transcript (Figure 3A). EGFR protein expression was also increased by CUL4A overexpression and decreased by CUL4A silence as evidenced by Western blot and IF (Figure 3B and C). Given the fact that EGFR expression is also correlated with poor prognosis in NSCLC [28], we examined the correlation between EGFR and CUL4A expression in tumors from patients with NSCLC. As expected, EGFR expression was found to be positively correlated with CUL4A level in lung cancer tissues (Figure 3D). Moreover, we verify the correlation between EGFR and CUL4A expression by analyzing tumors generated in nude mice (Additional file 6: Figure S6). These results indicate that CUL4A regulates the expression of EGFR.

**Figure 4:**
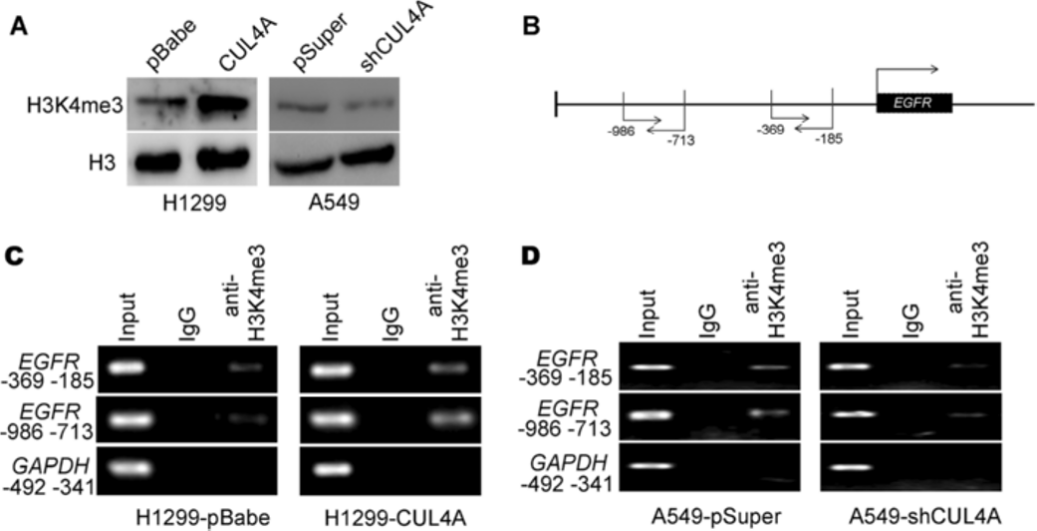
**CUL4A transcriptionally activates EGFR expression in NSCLC tissues. (A)** Western blot analysis of H3K4me3 levels in H1299-pBabe, H1299-CUL4A, A549-pSuper, and A549-shCUL4A cells. **(B)** Schematic presentation of two regions relative to the EGFR transcriptional start site used as primers to test H3K4me3 occupied abundance. **(C)** ChIP-PCR was performed to assess H3K4me3 occupancy in EGFR promoter in H1299-pBabe and H1299-CUL4A cells. **(D)** ChIP-PCR was performed to assess H3K4me3 occupancy in EGFR promoter in A549-pSuper and A549-shCUL4A cells. IgG was used as negative control.

Our previous study showed that CUL4A regulates histone methylation at H3K4 [29]. Thus, we proposed that CUL4A may transcriptionally activate EGFR expression through enrichment of H3K4 trimethylation (H3K4me3) at EGFR promoter. H1299 and A549 cells were used to verify our hypothesis. H1299-CUL4A cells showed higher level and A549-shCUL4A cells had lower level of H3K4me3 compared with their control cells (Figure 4A). ChIP assay was then performed using antibody against H3K4me3 and primers specific to EGFR promoter as indicated in Figure 4B. Our results indicated that the occupation of H3K4me3 at the EGFR promoter is significantly higher in H1299-CUL4A cells compared with H1299 cells with its control vector (Figure 4C). In contrast, silencing CUL4A gene expression in A549 significantly decrease the H3K4me3 occupation at the EGFR promoter compared with control cells (Figure 4D). These data collectively indicated that EGFR is transcriptionally activated by CUL4A expression through H3K4me3 modulation.

### CUL4A activates EGFR-mediated signaling pathways

**Figure 5:**
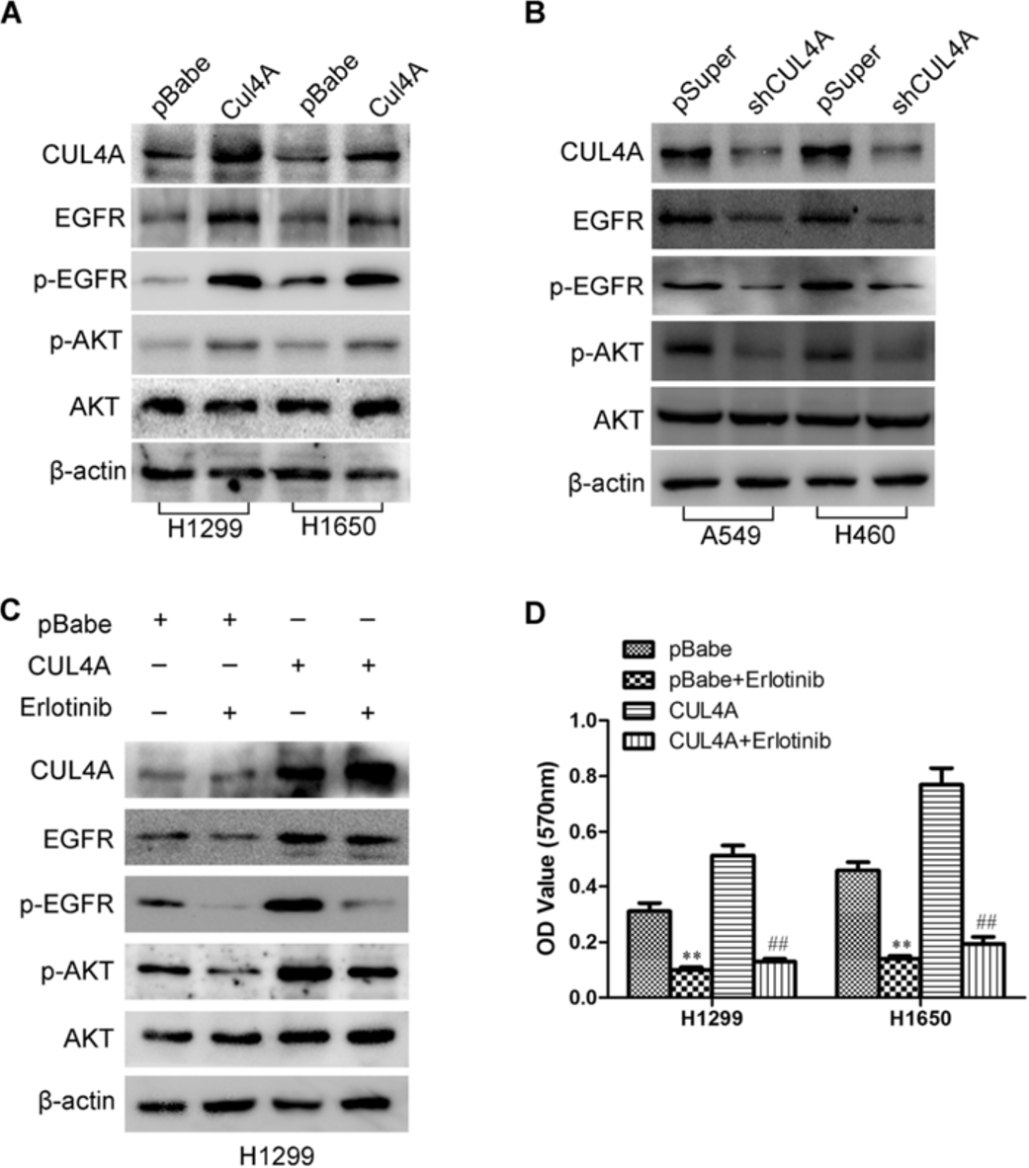
**CUL4A activates the EGFR-mediated signaling pathways. (A)** Levels of CUL4A, EGFR, p-EGFR, p-AKT, and AKT were analyzed by Western blot in H1299-pBabe, H1299-CUL4A, H1650-pBabe and H1650-CUL4A cells. **(B)** Levels of CUL4A, EGFR, p-EGFR, p-AKT, and AKT were analyzed by Western blot in A549-pSuper, A549-shCUL4A, H460-pSuper and H460-shCUL4A cells. **(C)** Western blot to analyze the effect of erlotinib on the levels of CUL4A, EGFR, p-EGFR, p-AKT, and AKT in H1299-pBabe and H1299-CUL4A cells. **(D)** MTT analysis of the inhibition of erlotinib on cell proliferation in CUL4A overexprssion cells (H1299-CUL4A and H1650-CUL4A). ^**^ *P* <0.01 *vs* pBabe cells; ^##^ *P* <0.01 *vs* pSuper cells. All results are from three independent experiments. Error bar indicate standard deviation.

Western blot showed that EGFR phosphorylation level altered in proportion to the change of total EGFR protein level when CUL4A expression is manipulated in H1299, H1650, A549 and H460 cells (Figure 5A and B), which indicates CUL4A may regulate the activation of EGFR signaling pathways in addition to total EGFR level. Thus, the phosphorylation and activation of EGFR downstream target proteins were analyzed. Western blot results showed that AKT phosphorylation was significantly increased by the overexpression of CUL4A although the total level of both AKT was not changed (Figure 5A), In contrast, silencing CUL4A led to inhibition of phosphorylation of AKT (Figure 5B).

To verify whether the activation of AKT by CUL4A in NSCLC cells is mediated through EGFR activation, H1299-CUL4A and its control cells were treated with erlotinib, an EGFR-tyrosine kinase inhibitor (EGFR-TKI), for 4 h. When EGFR phosphorylation was blocked by erlotinib, CUL4A induced AKT phosphorylation was reduced (Figure 5C).

To determine if the proliferative effect of CUL4A on NSCLC cells was EGFR dependent, we treated H1299-CUL4A, H1650-CUL4A and their control cells with erlotinib. Erlotinib clearly reduced the promotive effect of CUL4A on cell proliferation (Figure 5D). To evaluate whether CUL4A-EGFR-induced cell proliferation is due to upregulation of AKT signaling, we compared cell proliferation rates in H1299-CUL4A and its control cells in the presence and absence of inhibitor (LY294002) targeting PI3K. Treatment of the cells with 10 μM LY294002 blocked the induction of AKT phosphorylation (Additional file 7: Figure S7A). LY294002 also reversed proliferation of H1299 induced by CUL4A overexpression (Additional file 7: Figure S7B). These results suggest that Akt signaling activation is essential for CUL4A-induced proliferation.

Collectively, our data showed that CUL4A promotes NSCLC cell proliferation through EGFR-AKT pathway and high level of CUL4A expression sensitizes lung cancer cells to erlotinib.

## Discussion

To our knowledge, this is the first study to show that CUL4A has clinical significance and plays a functional role in human NSCLC. CUL4A was highly expressed in NSCLC and its expression was correlated with poor prognosis. Ectopic CUL4A expression in NSCLC cells induced proliferation and inhibited apoptosis *in vitro*. In contrast, silencing CUL4A reversed these events and resulted in inhibition of tumorigenic potential of NSCLC cells. We also verified a mechanistic link between CUL4A and EGFR through CUL4A mediated recruitment of H3K4me3 to EGFR promoter, which subsequently led to activation of EGFR expression and EGFR mediated signaling pathways. All of these functions of CUL4A conferred chemotherapy resistance and EGFR target therapy sensitivity to NSCLC cells.

Abnormal gene expression plays key roles in tumorigenesis which followed by series of target gene alterations and subsequent biological changes and this cascade of events is essential to tumorigenesis [30]. In addition to reported upregulation in breast carcinomas [16, 23], high level of CUL4A expression was also found in squamous cell carcinoma of the esophagus [31], Adrenocortical carcinoma [32], childhood medulloblastoma [33], hepatocellular carcinomas [17], malignant pleural mesothelioma [20] and prostate cancer [22]. In this study, we showed that CUL4A expression is frequently increased in human NSCLC tissues when compared with normal lung tissues and this elevation was significantly associated with NSCLC progression and prognosis. CUL4A is proposed as oncogenic based on its ability to ubiquitinate and degrade tumor suppressors, such as p21, p27, DDB2 and p53 [11–13, 34]. In this report we proposed a novel function of CUL4A in NSCLC. A serial evidence in our manuscript suggested that CUL4A activated EGFR transcription and its downstream signaling. EGFR signaling network plays a central role in the growth and maintenance of epithelial tissues, and alterations of this network can lead to malignant transformation [35, 36]. Overexpression of EGFR was found in 50-70% of human lung cancer [37], and deregulated expression of EGFR together with ligand binding and concomitant receptor activation promotes tumor cell growth, proliferation, and survival [38, 39]. Our current study found that the transactivating activity of EGFR could be stimulated by CUL4A upregulation and suppressed by CUL4A inhibition. In addition, CUL4A expression was found to be positively correlated with overexpression of EGFR in NSCLC patient tumors. However, the current report just tested the effects of CUL4A on EGFR expression and did not stratify the situation of EGFR gene amplification/ mutation. Such tests with the stratification of EGFR gene status will greatly expand the relevance of CUL4A to a broader population of EGFR overexpressing NSCLC tumors and will be explored in our future work.

Increased resistance to apoptosis is a hallmark alteration in most types of cancers [1]. Abrogation of proapoptotic pathways has been demonstrated to be one of the events key to tumor development and progression, and impairments in apoptotic programming are tightly linked to the commonly seen failure of anticancer chemotherapy and radiotherapy [40–42]. Thus, clarification of the mechanisms modulating the apoptosis/survival process in a particular cancer type will bring new insights in developing more effective therapeutic strategies. Notably, in the current study, we found that CUL4A plays an important role in antiapoptosis of NSCLC cells that is relatively insensitive to chemotherapy. Ectopic expression of CUL4A in NSCLC cells dramatically enhances their resistance to apoptosis induced by doxorubicin or docetaxel, two commonly used chemotherapeutics, whereas suppressing CUL4A expression with shRNA markedly abrogated the ability of NSCLC cells to resist cytotoxic reagent-induced cell death. Our results suggest that CUL4A contributs to sustaining the unwanted survival of NSCLC cells under the treatment of chemotherapeutics and targeting CUL4A may overcome chemotherapy resistance in NSCLC with high levels of CUL4A. In summary, our study demonstrates that NSCLC cells with CUL4A overexpression are relatively resistant to chemotherapy but sensitive to EGFR target therapy. Therefore, our experiments provide a good rational to believe that CUL4A is not only a potential therapeutic target, but also a therapeutic biomarker for sensitive to TKI and resistance to chemotherapy.

## Conclusions

In conclusion, we have identified a regulatory network of CUL4A-induced EGFR expression, which then targets AKT pathway to modulate cell growth of NSCLC. Our findings also suggest that CUL4A is not only a potential therapeutic target but may also serve as a novel prognostic and therapeutic biomarker for NSCLC.

## Methods

### Patients and specimens

This study was conducted with the approval of the Shandong University Institutional Ethical Review Board. Primary tumor specimens were obtained from 78 patients that underwent complete resection in Qilu Hospital of Shandong University between 2006 and 2008. Follow-up information was obtained from review of the patients’ medical record. None of the patients had received radiotherapy or chemotherapy before surgical resection. All 78 specimens were reevaluated with respect to histological subtype, differentiation, and tumor stage. The TNM staging system of the International Union Against Cancer was used to classify specimens as stages I (n =17), II (n =20), III (n =25), and IV (n =16). A total of 22 fresh tumor tissues and 22 fresh normal lung tissues were stored at -70°C immediately after resection for extraction of RNA.

### Cell lines

BEAS2B, HSAEpiC, A549, H1299, H460, A427, H1650, 95D, and HLAMP cell lines were from American Type Culture Collection (Manassas, VA). The cells were cultured in RPMI 1640 (Invitrogen, Carlsbad, CA) containing 10% fetal calf serum (Invitrogen), 100 IU/ml penicillin (Sigma, St. Louis, MO), and 100 μg/ml streptomycin (Sigma). Cells were grown on sterilized culture dishes and were passaged every 2 days with 0.25% trypsin (Invitrogen).

### Establishment of CUL4A stable expressing and knockdown cell lines

pBabe-puro retroviral constructs containing human *CUL4A* cDNA and pSuper.retro.puro with shRNA against human *CUL4A* cDNA were prepared as described previously [20]. The constructs were transfected into the HEK 293 Phoenix ampho packaging cells to produce retroviral supernatants. 48 h after transfection, the supernatant was filtered through a 0.25 μm syringe filter. Retroviral infection was performed by adding filtered supernatant to mammary cell lines in the presence of 8 μg/ml of polybrene (Sigma, St. Louis, MO, USA). 6 h after infection, medium was changed with fresh medium and infected cells were allowed to recover for 48 h. Infected cells were selected by adding 2 μg/ml puromycin (Sigma, St. Louis, MO, USA) to the culture medium for 48 h and then maintained in complete medium with 1 μg/ml puromycin. Empty retroviral-infected stable cell lines were also produced by the above protocols. The expression of CUL4A was confirmed by RT-PCR and Western blot analysis.

### Immunohistochemistry

Immunostaining was performed using the avidin-biotin-peroxidase complex method (UltrasensitiveTM, MaiXin, Fuzhou, China). The sections were deparaffinized in xylene, rehydrated with graded alcohol, and then boiled in 0.01 M citrate buffer (pH 6.0) for 2 min with an autoclave. Hydrogen peroxide (0.3%) was applied to block endogenous peroxide activity, and the sections were incubated with normal goat serum to reduce nonspecific binding. Tissue sections were incubated with CUL4A rabbit polyclonal antibody (1:250 dilution), EGFR mouse monoclonal antibody (1:150 dilution). Mouse immunoglobulin (at the same concentration of the antigen specific antibody) was used as a negative control. Staining for both antibodies was performed at room temperature for 2 h. Biotinylated goat antimouse serum IgG was used as a secondary antibody. After washing, the sections were incubated with streptavidin-biotin conjugated with horseradish peroxidase, and the peroxidase reaction was developed with 3, 30-diaminobenzidine tetrahydrochloride.

Two independent, blinded investigators examined all tumor slides randomly. Five views were examined per slide, and 100 cells were observed per view at 400× magnification. Scores for CUL4A and EGFR membrane and cytoplasmic staining were calculated based on staining intensity (0, below the level of detection; 1, weak; 2, moderate; and 3, strong) and the percentage of cells staining at each intensity level (0-100%). The final score was calculated by multiplying the intensity score by the percentage, producing a scoring range of 0 to 300. The immunohistochemistry score cut-off point was established as 73 using X-tile software program (version 3.6.3, Yale University School of Medicine, CT USA).

### RNA Extraction and semi-quantitative RT-PCR

Total cellular RNA was extracted from cells using the RNeasy Plus Mini Kit from (Qiagen). The quality and yield of the RNA samples were determined by ultraviolet spectrophotometer. Total RNAs (1 μg) were reverse transcribed to cDNA (20 μl) using PrimeScriptTM RT Kit (TaKaRa) according to the manufacturer’s instructions. PCR reaction was conducted with 2 μL cDNA sample, 0.4 μL forward primer (10 μmol/L), 0.4 μL reverse primer (10 μmol/L), 11.2 μL RNase-free water, and 6 μL 2× EsayTaq PCR SuperMix (TransGen BIotech, Beijing, China). PCR reaction was performed using the following cycle parameters: 95°C for 5 minutes, (94°C for 30 seconds, 56°C for 30 seconds, 72°C for 45 seconds) for 30 cycles, 72°C for 7 minutes. RT-PCR products were separated on 2% agarose gels. After stained with ethidium bromide, gel images were photographed with ChemiImagerTM 4400. RT-PCR was performed at least 3 times for each sample. The sequences of the primer pairs are:

CUL4A forward, 5′ ATACTTCAGGACCCACGTTTGAT 3′,

CUL4A reverse, 5′ TCTCCAAGTACTAAAGCAGGAAAATCT 3′,

EGFR forward, 5′ GCCACGTCTCCACACATCAG 3′,

EGFR reverse, 5′ TGGTGCATTTTCGGTTGTTG 3′,

GAPDH forward, 5′ ATAGCACAGCCTGGATAGCAACGTAC- 3′,

GAPDH reverse, 5′ CACCTTCTACAATGAGCT GCGTGTG 3′.

GAPDH was used as the reference gene.

### Western blot analysis

Total protein from cells was extracted in lysis buffer (Pierce) and quantified using the Bradford method. Then, 50 μg of protein were separated by SDS-PAGE (10%). After transferring to polyvinylidene fluoride (PVDF) membrane (Millipore, Billerica, MA), the membranes were incubated overnight at 4°C with antibodies against CUL4A (1:1000; CST), EGFR (1:1000; Abcam), β-actin (1:2000, Santa Cruz Biotechnology). After incubation with peroxidase-coupled antimouse IgG (Santa Cruz Biotechnology) at 37°C for 2 h, bound proteins were visualized using ECL (Pierce) and detected using BioImaging Systems (UVP Inc., Upland, CA). The relative protein levels were calculated based on beta-actin protein as a loading control.

### Soft agar assay

The test cells (3 × 10^5^) were suspended in 5ml of culture medium containing 0.4% agar (USB Corportion) and seeded onto a base layer of 5ml of 0.7% agar bed in 10-cm tissure-culture dishes. Colonies >50 μm in diameter were counted after 3 weeks.

### Confocal immunofluorescence microscopy

Cell lines were plated on culture slides (Costar, Manassas, VA, USA). After 24 hrs, the cells were rinsed with phosphatebuffered saline (PBS) and fixed with 4% paraformaldehyde in PBS, and cell membrane was permeabilized using 0.5% Triton X-100. These cells were then blocked for 30 min in 10% BSA (Sigma, Aldrich St. Louis, MO, USA) in PBS and then incubated with primary monoclonal antibodies in 10% BSA overnight at 4°C. After three washes in PBS, the slides were incubated for 1 hour in the dark with FITC-conjugated secondary goat anti-mouse, or goat anti-rabbit antibodies (Invitrogen, Grand Island, NY, USA). After three further washes, the slides were stained with 4-,6-diamidino-2-phenylindole (DAPI; Sigma, Aldrich St. Louis, MO, USA) for 5 min to visualize the nuclei, and examined using an Carl Zeiss confocal imaging system (LSM 780) ( Carl Zeiss, Jena, Germany).

### MTT assay

Cells were plated in 96-well plates in medium containing 10% FBS at about 3,000 cells per well 24 h after transfection. Then, 20 μl of 5 mg/ml MTT (Thiazolyl Blue) solution was added to each well and incubated for 4 h at 37°C, the media was removed from each well, and the resultant MTT formazan was solubilized in 150 μl of DMSO. The results were quantitated spectrophotometrically using a test wavelength of 570 nm.

### Apoptosis assay

Cells were harvested and washed twice with cold PBS by gentle shaking. Resuspend cells were added to Binding buffer and adjusted cell density to 2–5 × 10^5^/mL. In the dark, 5 μL Annexin V-FITC (50 mM TRIS, 100 mM NaCl, 1% BSA, 0.02% Sodium Azide, pH 7.4) was added to cell suspension Mix of 195 μL and incubated for 10 min at room temperature before adding 190 μL Binding buffer (1×) and 10μL PI. Ten thousand events per sample were acquired using a FACS-scan flow cytometer (Becton-Dickinson, San Jose, CA, USA) and the percentage of cell apoptosis were analyzed using Cell Quest analysis software (Becton-Dickinson).

### Chromatin immunoprecipitation assays

Cells were fixed in 1% formaldehyde for 10 minutes at 37°C. Cross-linking was quenched by adding 125 mmol/L glycine. Cells were then washed with cold PBS, harvested and resuspended in SDS lysis buffer containing a protease inhibitor cocktail. Chromatin was sheared by sonication (average length 0.25-1 Kb) and incubated with 60 ml protein A/G agarose/salmon sperm DNA (50% slurry; Millipore) with gentle agitation for 30 minutes. The supernatant was then immunoprecipitated with anti-SOX4 antibody 1:500 or its matched nonimmune crude serum 1:500 (IgG; Diagenode) at 4°C overnight. Protein A/G agarose (60 mL of 50% slurry) was then added and incubated for 1 hour. Pellets were washed and protein-DNA cross-links were reversed by overnight incubation at 65°C with proteinase K. DNA was purified following a conventional phenol–chloroform protocol and eluted in 50 mL water. At least 3 independent Chromatin immunoprecipitation (ChIP) experiments were carried out.

### Xenografted tumor model in vivo

Female BALB/c nude mice (4–5 weeks of age, 18–20 g) were purchased from the Center of Experimental Animal of Guangzhou University of Chinese Medicine and were housed in barrier facilities on a 12-hour light/dark cycle. All experimental procedures were approved by the Institutional Animal Care and Use Committee of Shandong University. The BALB/c nude mice were randomly divided into 2 groups (n =6/group). One group of mice were inoculated subcutaneously with A549/vector cells (1 × 10^6^, suspended in 100 μL sterile PBS) per mouse in the right oxter as control group. The other group was inoculated with A549/CUL4A shRNA cells (1 × 10^6^, suspended in 100 μL sterile PBS). Tumor volume was calculated using the equation (L × W^2^)/2.

### Statistical analysis

SPSS version 11.5 for Windows was used for all analyses. The ***χ***^2^ test was used to examine possible correlations between CUL4A expression and clinicopathologic factors. The association between CUL4A and EGFR immunointensity on the same specimens was analyzed using Spearman rank correlation test. The t test was used to compare data from the densitometry analysis of foci numbers. The Kaplan–Meier method was used to estimate the probability of patient survival, and differences in the survival of subgroups of patients were compared using Mantel’s log-rank test. A multivariate analysis was performed using the Cox regression model to study the effects of different variables on survival. *P* value of <0.05 was considered to indicate statistical significance.

## Electronic supplementary material

Additional file 1: Figure S1: CUL4A is overexpressed in lung cancer cell lines. (A) RT-PCR analysis of CUL4A mRNA levels in nine lung cell lines. (B) Western blot analysis of CUL4A protein levels in lung cancer cell lines. All experiments were repeated three times. Error bar indicate standard deviation. (JPEG 2 MB)

Additional file 2: Figure S2: CUL4A regulates NSCLC cell growth both *in vitro.* Cell proliferation *in vitro* was examined by MTT in H1650-pbabe, H1650-CUL4A (A) and H460-pSuper, H460-shCUL4A (B) cells. (JPEG 781 KB)

Additional file 3: Figure S3: CUL4A-induced lung cancer cell transformation *in vitro*. (A) Photomicrographs illustrating examples of soft agar colonies (left) and histobars indicating the statistical significance of the numbers of colonies (right) in H1299-pBabe and H1299-CUL4A cells. (B) Photomicrographs illustrating examples of soft agar colonies (left) and histobars indicating the statistical significance of the numbers of colonies (right) in A549-pSuper and A549-shCUL4A cells. ^**^ *P* <0.01. (JPEG 984 KB)

Additional file 4: Figure S4: The immunohistochemistry analysis of Ki67 expression in CUL4A-pBabe and CUL4A-shCUL4A cells xenograft tumors. Scale bar indicates 50 μm. (JPEG 777 KB)

Additional file 5: Figure S5: CUL4A regulated the sensitivity of NSCLC cells to chemotherapy. (A) MTT analysis of the viability of H1299 cell treated with different doses of doctaxel. (B) MTT analysis of the viability of H1299 cell treated with different doses of doxorubicin. (C) MTT analysis of the viability of H1650 cell treated with different doses of doctaxel. (D) MTT analysis of the viability of H1650 cell treated with different doses of doxorubicin. (E) MTT analysis of the viability of A549 cell treated with different doses of doctaxel. (F) MTT analysis of the viability of A549 cell treated with different doses of doxorubicin. (G) MTT analysis of the viability of H460 cell treated with different doses of doctaxel. (H) MTT analysis of the viability of H460 cell treated with different doses of doxorubicin. ^*^ *P* <0.05 and ^**^ *P* <0.01 *vs* pBabe cells; ^#^ *P* <0.05 and ^##^ *P* <0.01 *vs* pSuper cells. All results are from three independent experiments. Error bar indicate standard deviation. (JPEG 2 MB)

Additional file 6: Figure S6: The immunohistochemistry analysis of CUL4A and EGFR expression in CUL4A-pBabe and CUL4A-shCUL4A cells xenograft tumors. Scale bar indicates 50 μm. (JPEG 2 MB)

Additional file 7: Figure S7: LY294002 blocked the CUL4A-induced AKT phosphorylation and cell proliferation. Treatment of cells with 10 μM LY294002 blocked the induction of AKT phosphorylation (A). LY294002 also reversed proliferation of H1299 induced by CUL4A overexpression (B). ^**^ *P* <0.01 *vs* pBabe cells; ^##^ *P* <0.01 *vs* CUL4A cells. All results are from three independent experiments. Error bar indicate standard deviation. (JPEG 651 KB)

## Abbreviations

CUL4A: Cullin 4A
NSCLC: Non-small cell lung cancer
shRNA: Short hairpin RNA
FBS: Fetal bovine serum
PVDF: Polyvinylidene difluoride
TBST: Tris-buffered saline containing tween 20
BSA: Bovine serum albumin
ECL: Enhanced chemiluminescence
PBS: Phosphate-buffered saline
FACS: Fluorescence-activated cell sorting
ChIP: Chromatin immunoprecipitation.
