# Supplementary figures and images for: CUL4A overexpression enhances lung tumor growth and sensitizes lung cancer cells to Erlotinib via transcriptional regulation of EGFR
Source: Mol Cancer. 2014 Nov 21;13:252. doi: 10.1186/1476-4598-13-252 (PMC4246448; doi:10.1186/1476-4598-13-252)

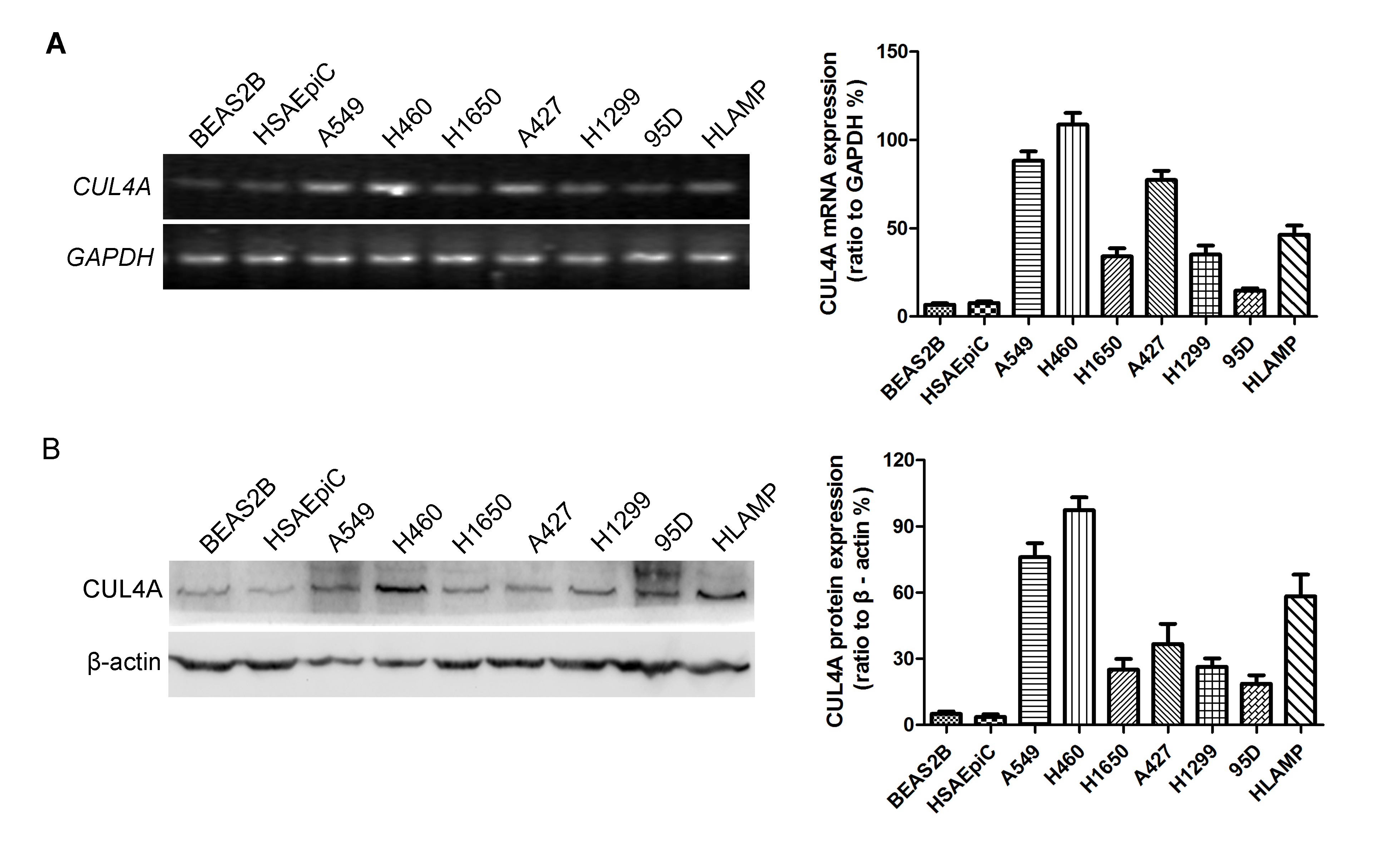

Supplement: Supplementary file 1 — Additional file 1: Figure S1: CUL4A is overexpressed in lung cancer cell lines. (A) RT-PCR analysis of CUL4A mRNA levels in nine lung cell lines. (B) Western blot analysis of CUL4A protein levels in lung cancer cell lines. All experiments were repeated three times. Error bar indicate standard deviation. (JPEG 2 MB) [file 12943_2014_1449_MOESM1_ESM.jpeg]

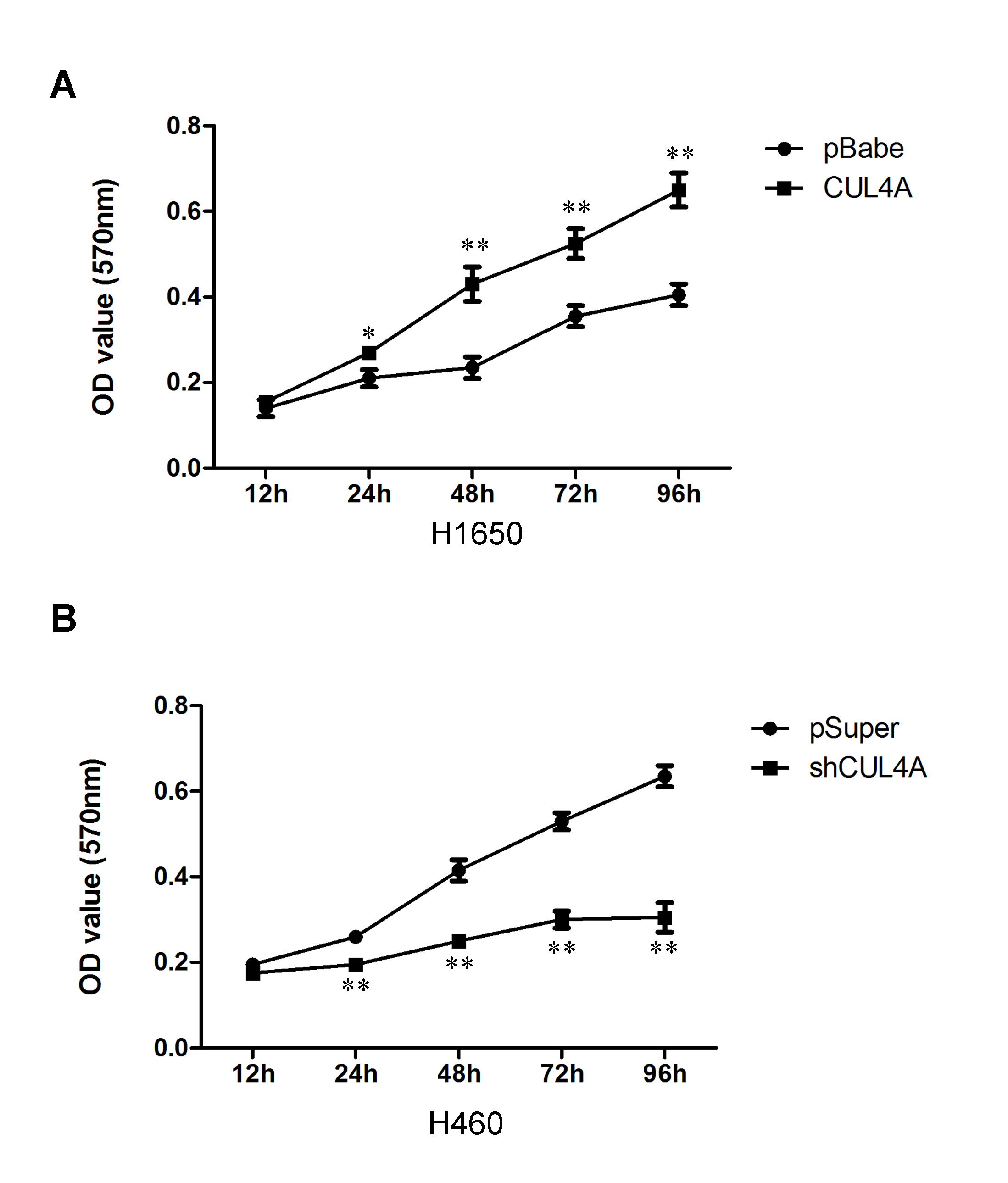

Supplement: Supplementary file 2 — Additional file 2: Figure S2: CUL4A regulates NSCLC cell growth both in vitro. Cell proliferation in vitro was examined by MTT in H1650-pbabe, H1650-CUL4A (A) and H460-pSuper, H460-shCUL4A (B) cells. (JPEG 781 KB) [file 12943_2014_1449_MOESM2_ESM.jpeg]

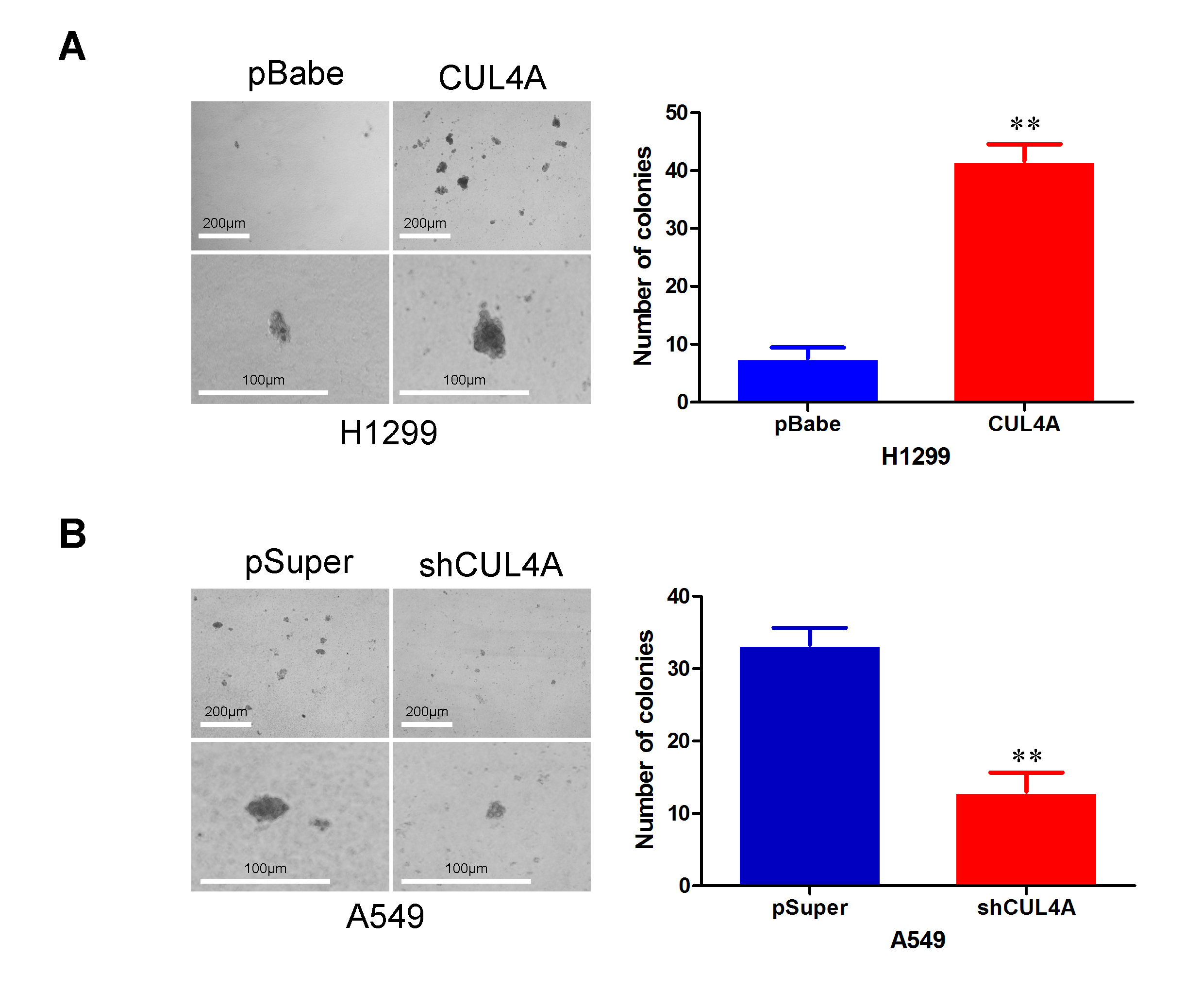

Supplement: Supplementary file 3 — Additional file 3: Figure S3: CUL4A-induced lung cancer cell transformation in vitro. (A) Photomicrographs illustrating examples of soft agar colonies (left) and histobars indicating the statistical significance of the numbers of colonies (right) in H1299-pBabe and H1299-CUL4A cells. (B) Photomicrographs illustrating examples of soft agar colonies (left) and histobars indicating the statistical significance of the numbers of colonies (right) in A549-pSuper and A549-shCUL4A cells. ** P <0.01. (JPEG 984 KB) [file 12943_2014_1449_MOESM3_ESM.jpeg]

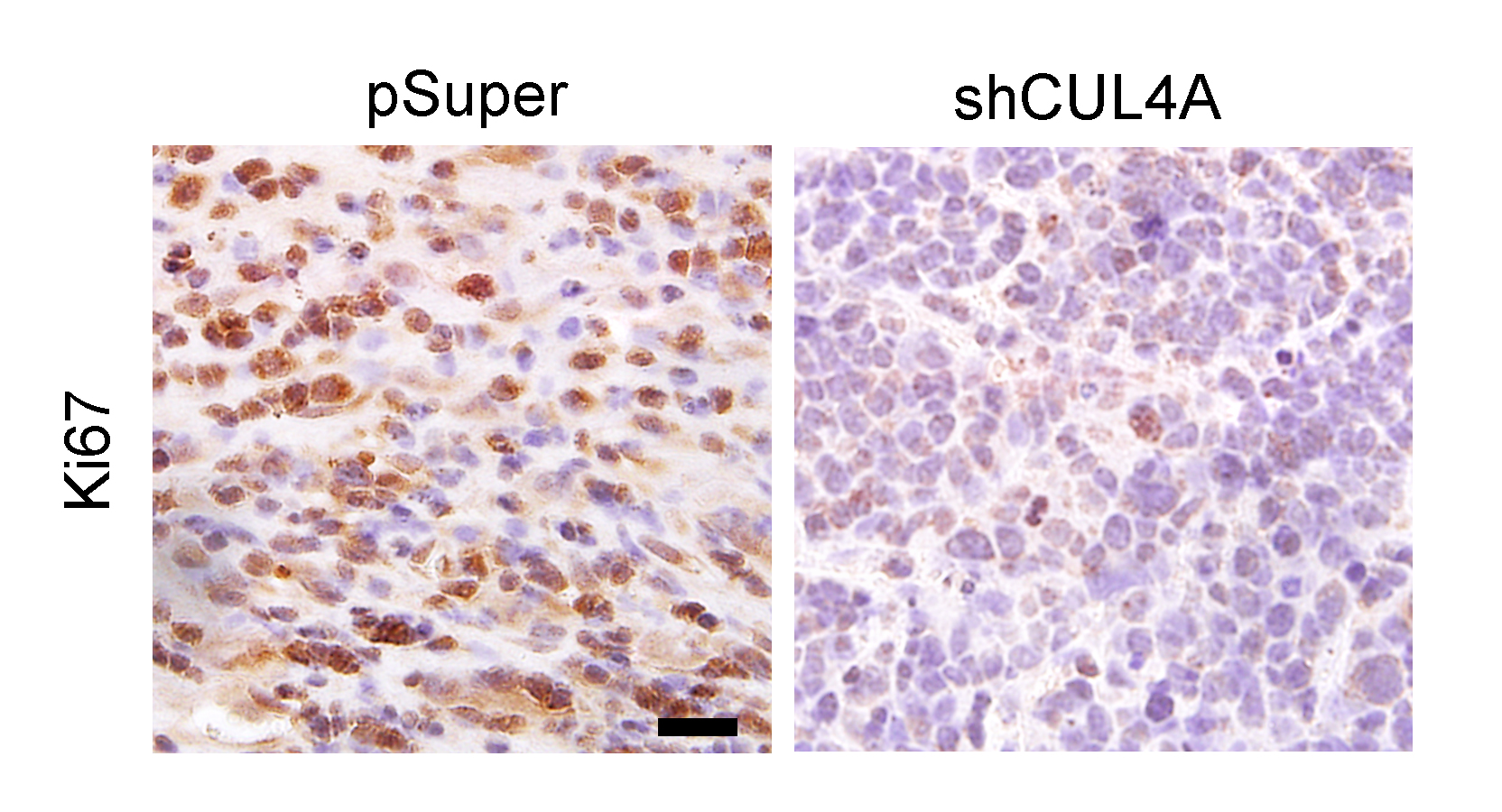

Supplement: Supplementary file 4 — Additional file 4: Figure S4: The immunohistochemistry analysis of Ki67 expression in CUL4A-pBabe and CUL4A-shCUL4A cells xenograft tumors. Scale bar indicates 50 μm. (JPEG 777 KB) [file 12943_2014_1449_MOESM4_ESM.jpeg]

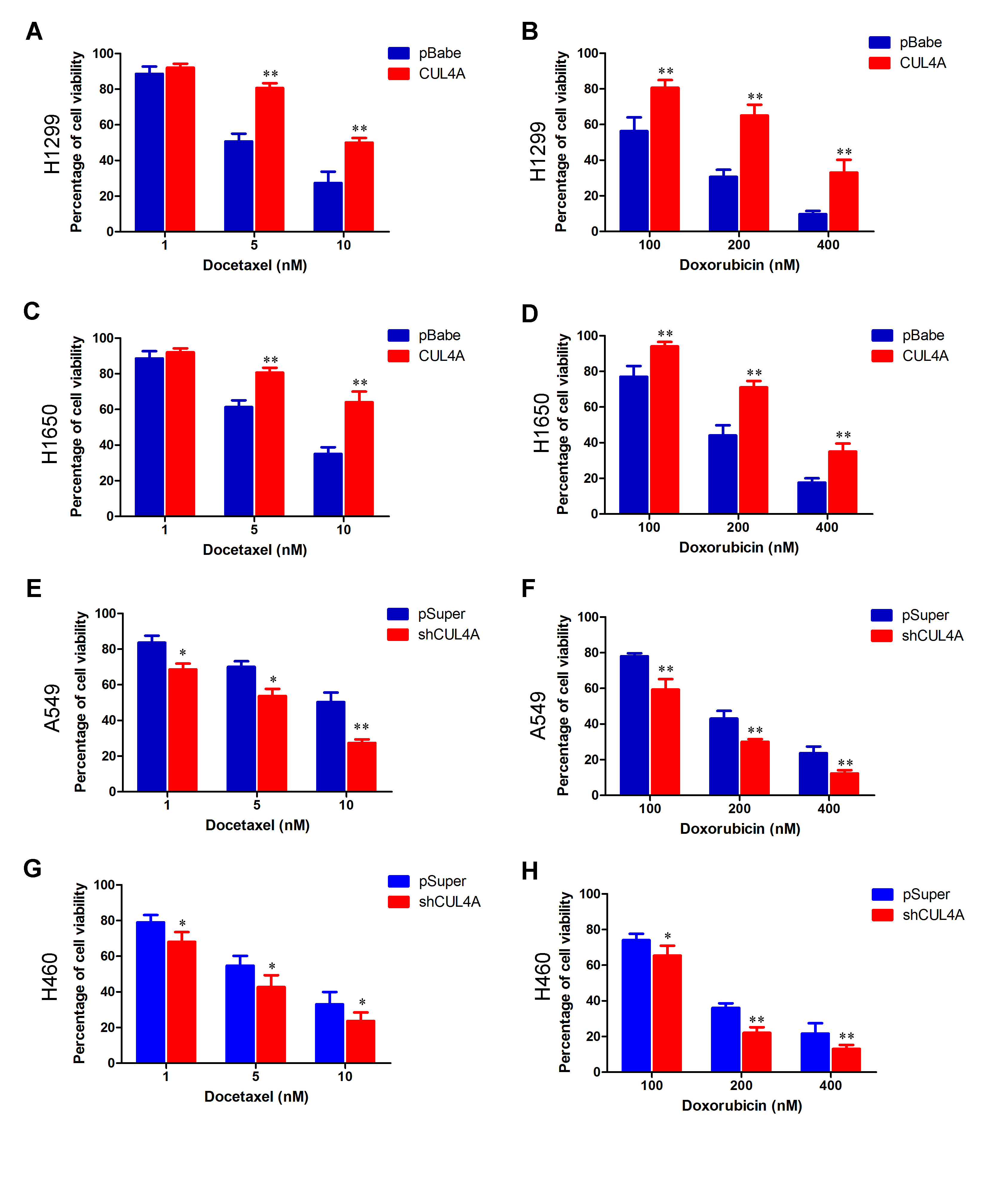

Supplement: Supplementary file 5 — Additional file 5: Figure S5: CUL4A regulated the sensitivity of NSCLC cells to chemotherapy. (A) MTT analysis of the viability of H1299 cell treated with different doses of doctaxel. (B) MTT analysis of the viability of H1299 cell treated with different doses of doxorubicin. (C) MTT analysis of the viability of H1650 cell treated with different doses of doctaxel. (D) MTT analysis of the viability of H1650 cell treated with different doses of doxorubicin. (E) MTT analysis of the viability of A549 cell treated with different doses of doctaxel. (F) MTT analysis of the viability of A549 cell treated with different doses of doxorubicin. (G) MTT analysis of the viability of H460 cell treated with different doses of doctaxel. (H) MTT analysis of the viability of H460 cell treated with different doses of doxorubicin. * P <0.05 and ** P <0.01 vs pBabe cells; # P <0.05 and ## P <0.01 vs pSuper cells. All results are from three independent experiments. Error bar indicate standard deviation. (JPEG 2 MB) [file 12943_2014_1449_MOESM5_ESM.jpeg]

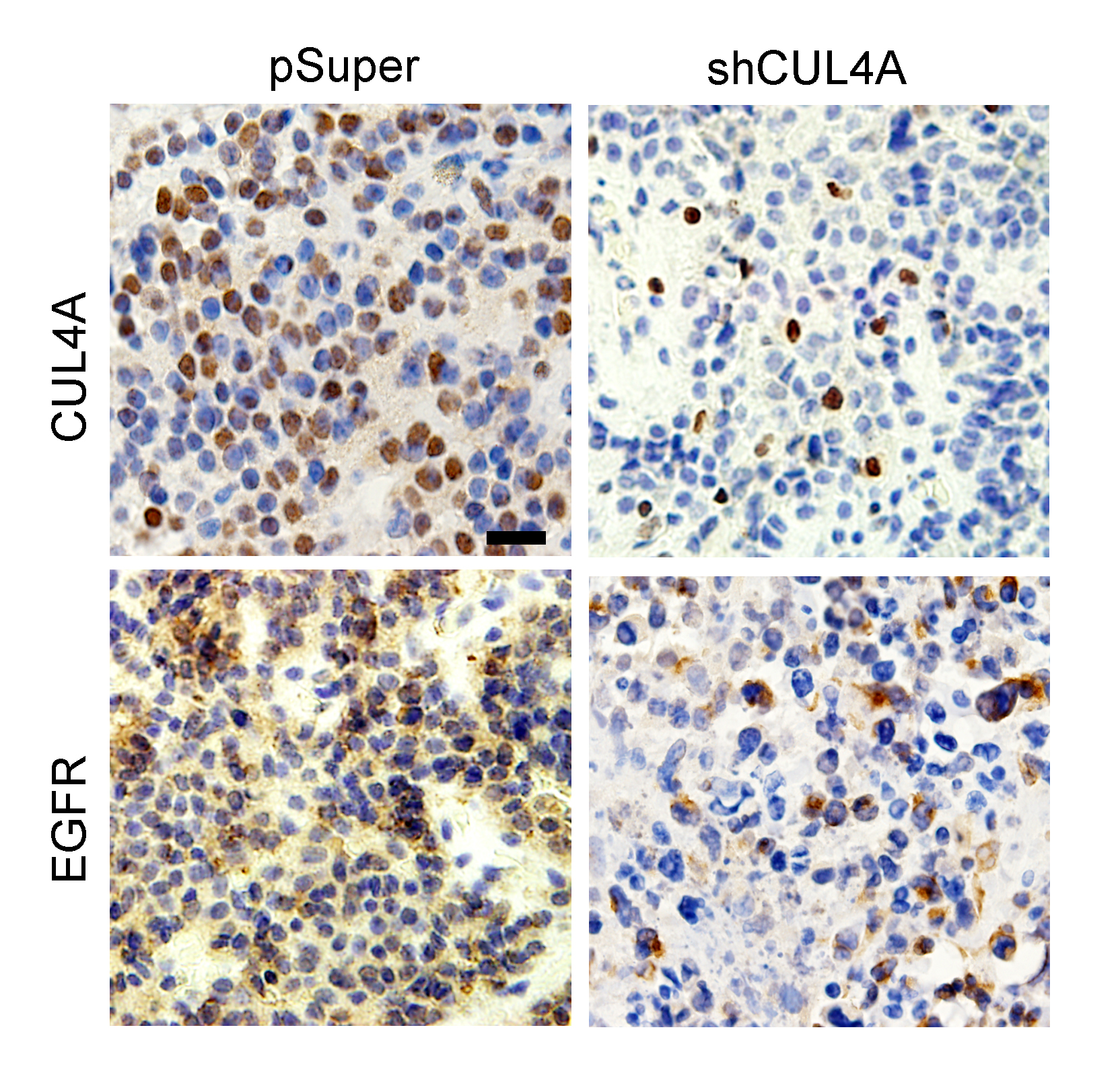

Supplement: Supplementary file 6 — Additional file 6: Figure S6: The immunohistochemistry analysis of CUL4A and EGFR expression in CUL4A-pBabe and CUL4A-shCUL4A cells xenograft tumors. Scale bar indicates 50 μm. (JPEG 2 MB) [file 12943_2014_1449_MOESM6_ESM.jpeg]

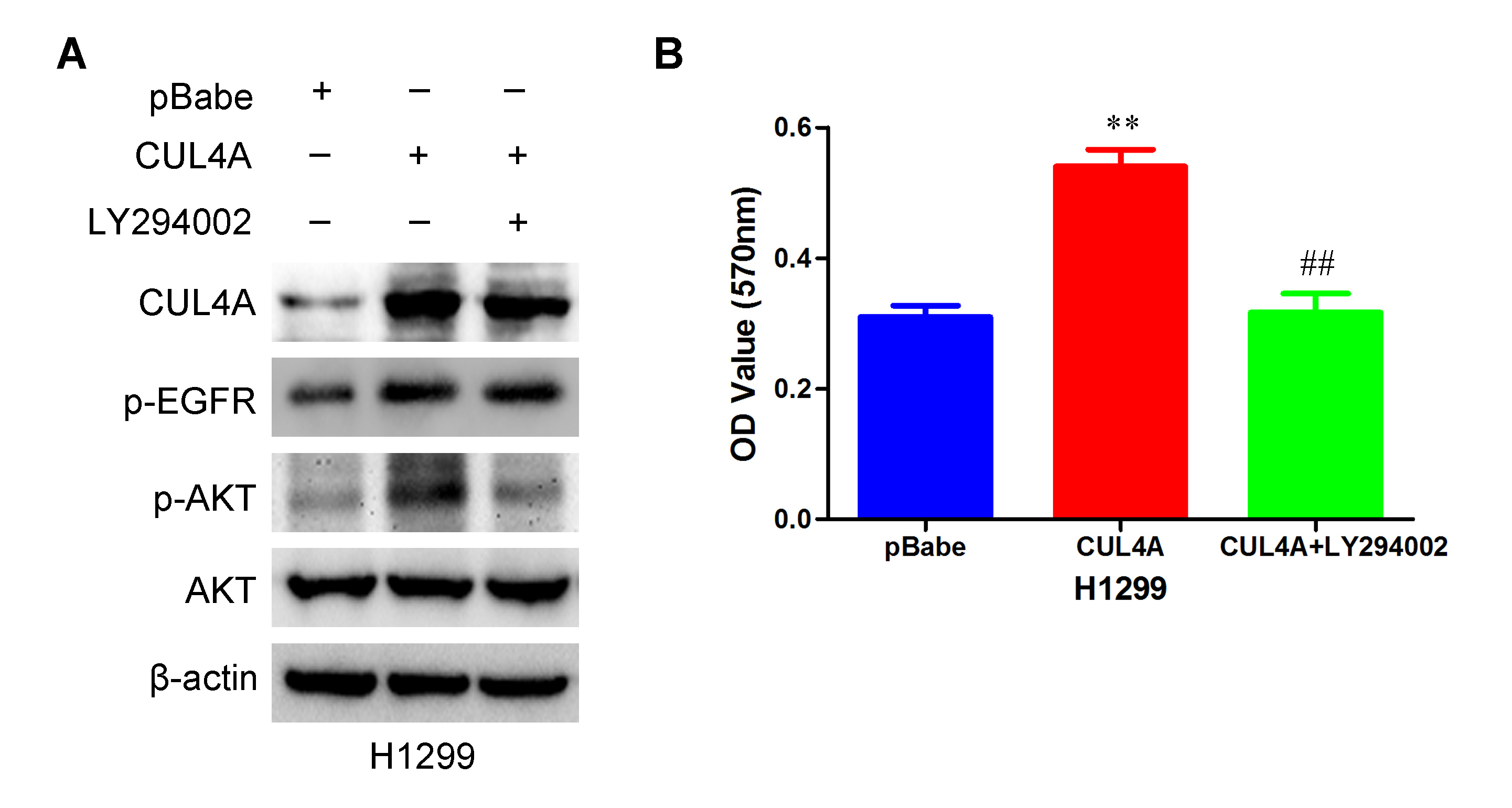

Supplement: Supplementary file 7 — Additional file 7: Figure S7: LY294002 blocked the CUL4A-induced AKT phosphorylation and cell proliferation. Treatment of cells with 10 μM LY294002 blocked the induction of AKT phosphorylation (A). LY294002 also reversed proliferation of H1299 induced by CUL4A overexpression (B). ** P <0.01 vs pBabe cells; ## P <0.01 vs CUL4A cells. All results are from three independent experiments. Error bar indicate standard deviation. (JPEG 651 KB) [file 12943_2014_1449_MOESM7_ESM.jpeg]
